# Severe Cardiomyopathy after Huffing Dust-Off*™*

**DOI:** 10.1155/2016/9204790

**Published:** 2016-05-25

**Authors:** Alexis L. Cates, Matthew D. Cook

**Affiliations:** ^1^William Carey University College of Osteopathic Medicine, Hattiesburg, MS 39401, USA; ^2^Department of Emergency Medicine, Lehigh Valley Hospital and Health Network/USF MCOM, Allentown, PA 18017, USA

## Abstract

A 34-year-old man was found down in a parking lot after huffing fifteen cans of Dust-Off. Though lucid during the initial hospital evaluation, the patient experienced a generalized seizure followed by a torsades de pointes arrhythmia and was resuscitated. An echocardiogram revealed left and right ventricular dysfunction with an ejection fraction of 25%. This unique outcome of inhalant abuse has scarcely been reported in similar cases. The patient fully recovered and had a normal ejection fraction prior to discharge.

## 1. Background

Huffing, or inhalant abuse, is known to cause respiratory distress, cough, hypoxia, nausea, vomiting, central nervous system (CNS) depression, and myocardial sensitization [1]. Dust-Off is a refrigerant-based propellant cleaner used for removal of dust and debris from keyboards, screens, and other electronics [2]. The active ingredient is difluoroethane, a clear, colorless, liquefied hydrocarbon gas [2]. When inhaled, this product “is harmful and may cause heart irregularities, unconsciousness, or death” [2, 3]. The material safety data sheet for Dust-Off notes that higher exposure may induce temporary palpitations, an irregular pulse, or inadequate circulation [2]. Other adverse effects include simple asphyxia and frostbite [2].

## 2. Case Presentation

**Figure 1:**
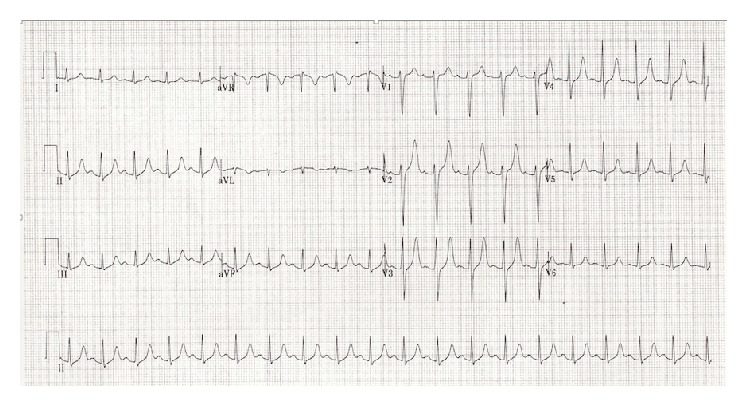
ECG displaying sinus tachycardia.

A 34-year-old white male with a history of depression and seizure disorder was found down by police with fifteen empty cans of Dust-Off nearby. He was arousable on scene and was transported by the Emergency Medical Services to a local Emergency Department, where he remained awake and alert. He admitted to huffing the Dust-Off, as well as a history of recurrent and heavy hydrocarbon huffing. An electrocardiogram (ECG) was obtained and revealed sinus tachycardia with a prolonged QTc of 472 milliseconds (Figure 1). The patient was transferred to a regional burn unit for frostbite on his hand due to refrigerant exposure.

**Figure 2:**
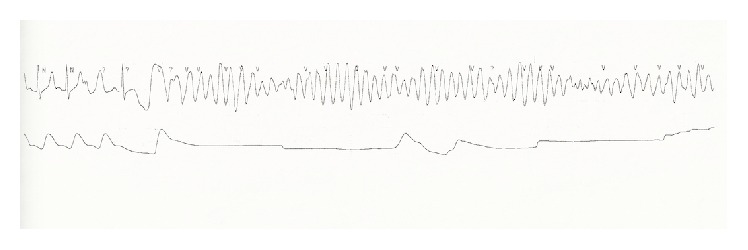
Rhythm strip showing torsades de pointes.

While in the burn unit, the patient had a generalized seizure and went into cardiac arrest. Advanced Cardiac Life Support protocol was initiated. The patient was found to be in a torsades de pointes rhythm (Figure 2) and was given magnesium. He was intubated, resuscitated, and sent to the Cardiac Intensive Care Unit where he was placed on fentanyl and midazolam drips and continuously monitored. Episodes of hypotension and tachycardia were noted, as well as right bundle branch block on repeat ECG. He was started on multiple vasopressors and given amiodarone for dysrhythmias. Hypocalcemia (5.6 mg/dL) was treated with calcium gluconate boluses and resolved.

Pertinent medical history included multiple psychiatric admissions and tobacco and prior alcohol abuse. The patient was homeless and unemployed. Possible outpatient medications included multiple antidepressants and sedatives, but compliance was unknown.

At the time of toxicology consultation approximately 24 hours after presentation, physical exam revealed a young white male, intubated, arousable but easily agitated. Vital signs included a heart rate of 106, blood pressure of 110/67, respiratory rate of 14 per minute, and 100% oxygen saturation on the ventilator. Maximum temperature was noted to be 103.5 degrees Fahrenheit, with a temperature of 99.3 degrees Fahrenheit at consultation. His pupils were 3 mm and reactive to light. He was tachycardic with a regular rhythm. The remainder of the exam was benign with no appreciable neurological deficits, rigidity, or clonus.

Initial chemistry panel revealed creatinine of 1.55 mg/dL, sodium of 138 mmol/L, chloride of 103 mmol/L, bicarbonate of 16 mmol/L, potassium of 3.5 mmol/L, magnesium of 3.0 mg/dL, and an anion gap of 19. AST was mildly elevated at 64 U/L with an ALT of 48 U/L. Arterial blood gas analysis revealed pH of 7.38, pCO_2_ of 37 mmHg, pO_2_ of 80 mmHg, HCO_3_ of 22 mEq/L, and oxygen saturation at 94% prior to intubation. Over the next 24 hours, the patient was noted to have elevated lactate of 4.7 mmol/L and evidence of rhabdomyolysis (maximum CK 5058 U/L, day 2), shock liver (maximum AST 17432 U/L, day 3), acute kidney injury (maximum creatinine 1.86 mg/dL, day 2), troponin elevation (maximum 20.20 ng/mL, day 2), and coagulopathy (maximum INR 3.0, day 2).

Echocardiogram revealed left and right ventricular dysfunction with an ejection fraction of 25% and global hypokinesis. Multiple chest X-rays were negative for an acute cardiopulmonary process. Labs drawn prior to discharge revealed resolving transaminitis and rhabdomyolysis, as well as normalized renal function and INR.

The patient was discharged eight days after admission with a repeat echocardiogram showing normal left ventricle size and function, normal wall motion, and an ejection fraction of 55%. He confirmed that he had only used Dust-Off on the day of presentation.

## 3. Discussion

This unusual presentation of a rapidly resolved cardiomyopathy after inhalation of several cans of Dust-Off shows the consequential cardiac effects of difluoroethane. Animal studies and case reports have reported that halogenated hydrocarbons will sensitize the myocardium to catecholamines, which may induce an arrhythmia and result in hypertrophied cardiac muscle [4]. However, a resolved case of such severe cardiac pathology is rarely reported.

A similar case of a 20-year-old male reports acute ingestion of alprazolam and oxycodone tablets preceded by inhalation of Dust-Off 2 to 3 days earlier [4]. Diffuse ST-T changes were noted on ECG and left ventricular dysfunction was seen on echocardiogram with an ejection fraction of 10–15% [4]. Sensitization of the myocardium by difluoroethane made the patient more susceptible to catecholamine-induced cardiomyopathy [4].

A case of a 42-year-old male with repeated exposure to 1,1-difluoroethane contained in CRC Duster reported the cause of death to be a fatal cardiac arrhythmia. Autopsy revealed arteriosclerotic coronary artery disease, including an acute infarction of the left ventricle [5].

Torsades de pointes and transient cardiomyopathy following huffing intoxication with Dust-Off is a rare finding. Emergency physicians taking care of patients with acute halogenated hydrocarbon toxicity should be aware of the potential cardiac effects and monitor the patients for cardiomyopathies and arrhythmias. Serial ECGs and echocardiogram are suggested when indicated. Antiarrhythmic and supportive measures should be initiated as indicated.
